# Early feeding strategies in lambs affect rumen development and growth performance, with advantages persisting for two weeks after the transition to fattening diets

**DOI:** 10.3389/fvets.2022.925649

**Published:** 2022-07-28

**Authors:** Ting Liu, Fadi Li, Weimin Wang, Xiaojuan Wang, Zhiyuan Ma, Chong Li, Xiuxiu Weng, Chen Zheng

**Affiliations:** ^1^College of Animal Science and Technology, Gansu Agricultural University, Lanzhou, China; ^2^State Key Laboratory of Grassland Agro-Ecosystems, College of Pastoral Agriculture Science and Technology, Lanzhou University, Lanzhou, China

**Keywords:** early supplementation, early weaning, lamb, rumen development, growth performance

## Abstract

This study aimed to explore the effects of early feeding strategies on the growth and rumen development of lambs from pre-weaning to the transition to fattening diets. Ninety-six newborn, male lambs with similar body weights were randomly assigned to three treatments: fed starter at 42 days old + weaned at 56 days old (Ctrl*, n* = 36), fed starter at 7 days old + weaned at 56 days old (ES, *n* = 36), and fed starter at 7 days old + weaned at 28 days old (ES + EW, *n* = 24). The fattening diets of all lambs were gradually replaced from 60 to 70 days of age. Six randomly selected lambs from each treatment were slaughtered at 14, 28, 42, 56, 70, and 84 days of age. The results showed that the richness and diversity of rumen microbiota of lambs in the Ctrl group were distinct from those of lambs in the other groups at 42 days of age. Moreover, transcriptome analysis revealed 407, 219, and 1,211 unique differentially expressed genes (DEGs) in the rumen tissue of ES vs. Ctrl, ES vs. ES + EW, and ES + EW vs. Ctrl groups, respectively, at 42 days of age. Different early feeding strategies resulted in differences in ruminal anatomy, morphology, and fermentation in lambs from 42 to 84 days of age (*P* < 0.05). Lambs in the ES + EW group had a higher average starter diet intake than those in the other groups (*P* < 0.05) from 28 to 56 days of age, which affected their growth performance. After 42 days of age, the body and carcass weights of lambs in the ES and ES + EW groups were higher than those in the Ctrl group (*P* < 0.05). These findings demonstrate that feeding lambs with a starter diet at 7 days of age and weaning them at 28 days of age can promote rumen development and improve growth performance, and this advantage persists for up to 2 weeks after transition to the fattening diet.

## Introduction

Rumen development is the most critical physiological challenge in young ruminants. The ruminant forestomach has a unique structure and function that not only differentiates it from the stomach of monogastric animals morphologically but also in terms of digestion and metabolism (1). Lambs are born with a physically and metabolically underdeveloped rumen similar to that of a monogastric animal, whereby the milk enters the abomasum through the esophageal groove to be digested and is then absorbed in the small intestine to maintain and meet the nutrient requirements for growth (2, 3). After lambs start consuming solid feeds, the rumen's colonization by microorganisms, establishment of fermentation, initiation of transport and absorption, volume enlargement, and growth of papillae are all necessary as lambs shift from dependence on milk to solid feeds (4, 5). Numerous studies have confirmed that even subtle changes in early feeding styles and nutrient composition are able to substantially influence ruminal development (6–9).

The weaning age of lambs is generally determined by the production goal in the commercial lamb industry. Early weaning can shorten the breeding cycle of ewes and be a strong stressor for lambs, as separation from the dams increases lamb morbidity and mortality (10). Lactation time is an important factor in ewe-lamb contact intensity and early weaning stress (11). Furthermore, early weaning success is limited by rumen volume, the rate of functional development (12, 13), and the establishment of a functional rumen microbiome (14, 15). Studies related to the early weaning of lambs have mainly focused on growth performance (16, 17), rumen development (9), and rumen microbiota (18, 19). Therefore, no uniform standard for the early weaning of lambs has been established so far and a weaning strategy that minimizes the negative impact of early weaning while maintaining economic benefits is required.

Early supplementation has been shown to improve rumen development and function during the transition from milk to solid feeds (20, 21), preventing several issues related to weaning. Previous studies have confirmed a positive correlation between starter diet intake and rumen development (9, 22), as well as diet fermentability (23) and feed additives (24). Similarly, the levels and physicochemical characteristics of the available substrates affect rumen microbial diversity and fermentation patterns after lambs begin to consume solid feeds (25, 26). For example, feeding lambs readily fermentable carbohydrates, including starch and sugar, increases volatile fatty acids (VFAs), especially butyrate, which is responsible for ruminal epithelial development (27). Additionally, feeding forage-containing starter diets stimulates rumen muscularization and rumination, enhances rumen volume and motility, and maintains rumen wall integrity and health (28). Thus, decades of research has focused on feeding strategies to facilitate early weaning and the transition from liquid to solid feeds in lambs (29–32). Abecia et al. (33) showed that nutritional intervention in young goats during the pre-weaning period can influence the rumen microbial composition, with the effects lasting for 3 months after weaning. However, there are no published studies characterizing the long-term effects of pre-weaning feeding strategies on lambs. We hypothesized that feeding strategies during the pre-weaning period of lambs would influence rumen development, affecting the ruminal microbiota and ruminal epithelium to change the ruminal fermentation pattern and function. This would occur *via* changes in protein and gene expression, and the ruminal microbiota, with the effects persisting after weaning. Therefore, the present study aimed to investigate the effects of lambs' early feeding strategies on rumen development and performance from pre-weaning to transition to fattening diets.

## Materials and methods

### Animal management and diets

Ninety-six newborn male lambs (3.58 ± 0.66 kg) from Hu ewes with double male lambing were obtained from a commercial sheep farm in Gansu Province, China. The ewes and lambs were housed in well-ventilated sheep pens (6 m × 8 m) with eight lambs and their dams per pen. All ewes were fed three times daily according to the feeding management of the sheep farm and had *ad libitum* access to water. The ewes were fed a total mixed feed of 23.90% corn silage, 15.41% oat hay, 12.84% alfalfa hay, 10.35% barley straw, 6.34% oilseed rape straw, 16.10% soybean meal, 11.30% corn, and 3.75% soybean meal. When the ewes were fed, lambs were separated from their dams and housed in individual pens (2.5 m × 1.5 m); however, the lambs and their dams could see each other. Throughout the experimental period, the lambs were unable to feed on the ewes' diet.

**Figure 1:**
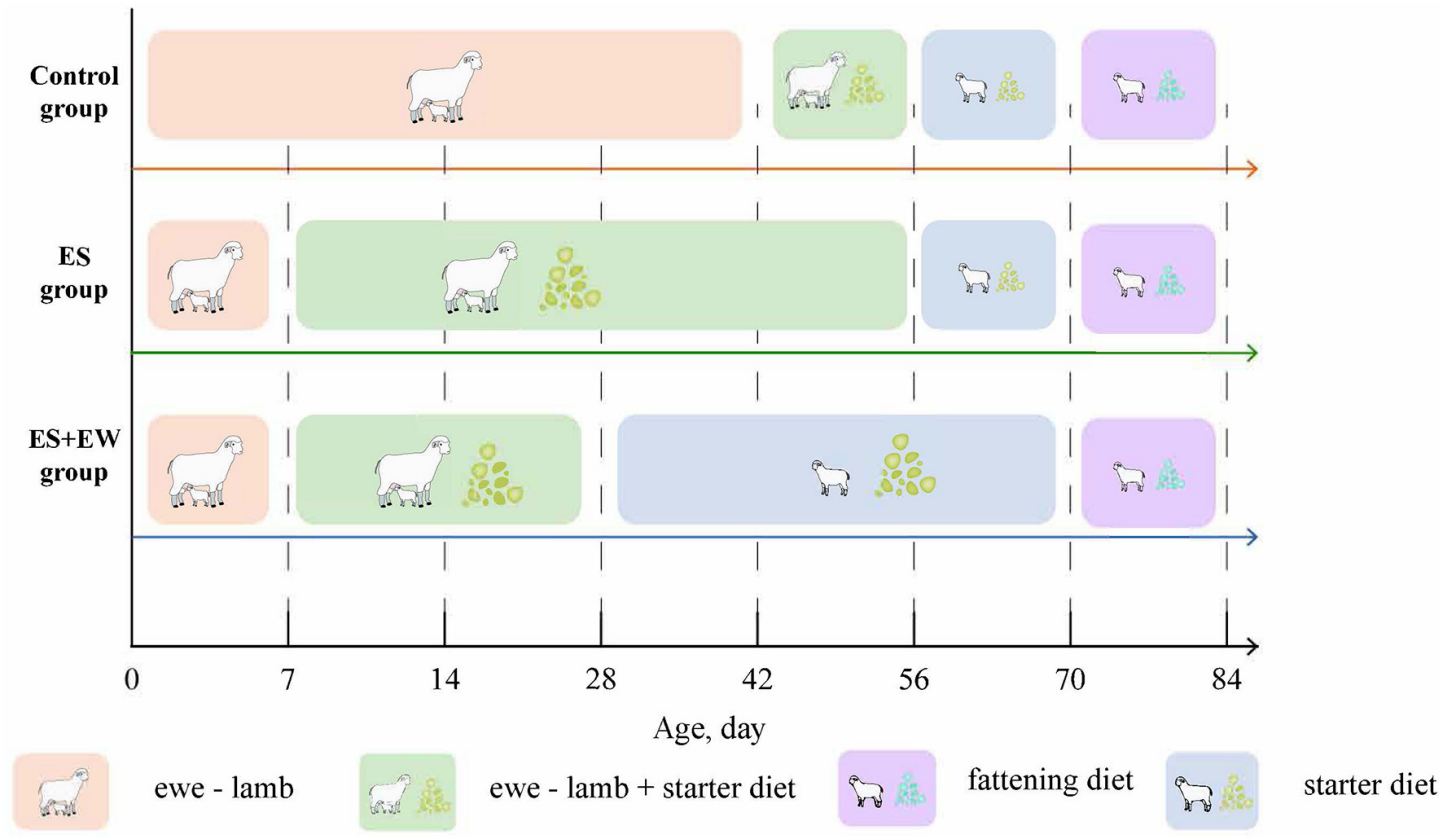
Experimental design. Study design showing the time frame of the experiment and the treatment groups. The ewes of control group were fed according to the sheep farm procedure. At 42, 56, 70, and 84 days of age, six lambs were randomly selected from each treatment group, weighed, and slaughtered.

As described in Figure 1, when the lambs were 7 days old, 36 lambs (birth weight, BW = 4.94 ± 0.80 kg) were randomly selected as the control group (Ctrl); these lambs were raised with ewes, fed on starter diet at 42 days old, and weaned at 56 days old. Other lambs (*n* = 60; 7 days old; BW = 4.91 ± 0.81 kg) were offered a pelleted starter diet *ad libitum* during the time they were separated from their dams (the starter was offered each morning and the residue was collected the next morning). Twenty-four of these lambs (28 days old; BW = 7.58 ± 1.62 kg) were weaned at 28 days of age and categorized as the early weaning group (EW + ES). The remaining lambs (*n* = 30; 28 days old; BW = 8.38 ± 0.96 kg) served as the early supplementary group (ES). The lambs in the Ctrl and ES groups were weaned at 56 days of age. After weaning, the lambs were housed in pens (6 m × 8 m) where they could not see the dams. The fattening diets of all lambs were gradually replaced from 60 to 70 days of age. The lambs had *ad libitum* access to the experimental diets and water during the experimental period. Six lambs were selected from each group and slaughtered on days 14, 28, 42, 56, 70, and 84.

The experimental diets were formulated and calculated according to the Nutrient Requirements of Small Ruminants (34). The experimental diets were provided as 2.5 mm pellets. Table 1 shows the experimental diets, including their formulation and chemical composition. The feedstuffs were analyzed for dry matter (DM, method 930.15), crude protein (CP, method 984.13), calcium (method 975.03), and phosphorous (method 965.17) according to the Association of Official Agricultural Chemists (AOAC) methods (35), while starch content was determined according to the method described by MacRae et al. (36). The determination of neutral detergent fiber (NDF) and acid detergent fiber (ADF) were based on the method of Van Soest et al. (37). All analyses were performed in triplicate. The digestive energy (DE) was computed according to the National Research Council (NRC) methodology (34) using sheep tabular values.

**Table 1:** Ingredients and chemical composition of experimental diet (%, DM basis).

**Item**	**Starter diet**	**Fattening diet**
Ingredients		
Alfalfa hay	5.0	25.0
Corn	55.9	44.5
Soybean meal	11.0	9.0
Whey powder	1.5	–
Expended soybean	7.0	–
Dried barley malt rootlets	17.0	18.0
Limestone	1.2	0.7
Premix^a^	1.0	1.0
NaCl	0.3	0.4
Feed attractant	0.1	–
NaHCO_3_	–	1.4
Nutrient composition, % of dry matter^b^		
DM, air dry basis	87.5	87.6
DE (MJ/kg)	13.5	12.1
CP	18.2	14.6
NDF	18.0	22.0
Starch	38.8	28.9
Calcium	0.7	0.7
Phosphorus	0.3	0.4

a *Premix provides the following minerals and vitamins per kg diet: S, 2 g/kg; Fe, 38 mg/kg; Zn, 23 mg/kg; Cu, 8.7 mg/kg; I, 0.77 mg/kg; Mn, 20 mg/kg; Se, 0.14 mg/kg; Co, 0.16 mg/kg; vitamin A, 1,566 IU/kg; vitamin D 220 IU/kg; vitamin E 21 IU/kg*.

b *DM, CP, Starch, NDF, Calcium, and Phosphorus were measured values, while the others were calculated values*.

### Sample collection

Samples were collected as described in Liu et al. (26). In brief, the body weight of each lamb was determined weekly from 7 to 84 days of age. The starter and fattening dietary intakes were measured daily for each pen. For each lamb, diarrhea was monitored and recorded daily to calculate the diarrhea rate. At 42, 56, 70, and 84 days of age, six lambs were randomly selected from each treatment group, weighed, and slaughtered in the abattoir. Immediately after slaughter, both carcass and organ weight were measured. The entire rumen content was collected, homogenized, and a subsample was transferred into a plastic bottle, snap-frozen in liquid nitrogen, and stored at −80°C for bacterial analysis. Ruminal tissues from the ventral sac region were sampled and stored at −80°C for transcriptomic analysis or washed in normal saline and stored in 10% buffered formalin solution for morphological analysis. Ruminal fluid was collected and stored at −20°C for the determination of volatile fatty acids (VFAs), ammonia nitrogen (NH_3_-N), and microbial crude protein (MCP) concentrations.

### Measurement of physiological parameters

Immediately after slaughter, rumen pH was determined using a pH meter (Orion Star A121 portable pH meter; Thermo Fisher Scientific, Waltham, MA, USA). The concentrations of VFAs, NH_3_-N, and MCP in the ruminal fluid were analyzed as described previously (26, 38, 39). In brief, VFAs were measured in an Agilent 6890N gas chromatograph (Agilent Technologies Inc., Santa Clara, CA, USA) with a 30 m (0.32 mm internal diameter) fused silica column (HP-19091N-213; Agilent Technologies Inc.), as described by Liu et al. (26). The NH_3_-N content was measured using a SP-723 spectrophotometer (Spectrum Instruments, Ltd., Shanghai, China) according to the Berthelot reaction (phenol-hypochlorite), as described by Broderick and Kang (38). The MCP content was measured using the Folin phenol method, as described by Makkar et al. (39). Ruminal morphometric analyses were performed using hematoxylin-eosin staining, as described by Lesmeister et al. (40).

### DNA extraction, 16S rRNA amplicon sequencing, and bacterial composition analysis

For microbial DNA extraction, rumen contents were collected from six lambs in each group at 42 days of age. Total DNA was extracted from rumen contents using a QIAamp DNA Stool Mini Kit (QIAGEN, Hilden, Germany), according to the manufacturer's instructions. To assess the rumen microbial profiles, the V3–V4 region of the bacterial *16S rRNA* gene was amplified using the universal primers 341F (5′-CCTAYGGGRBGCASCAG-3′) and 806R (5′-GGACTACNNGGGTATCTAAT-3′) (41) with barcode sequences. The obtained amplicons libraries for all samples were sequenced on the Illumina MiSeq platform (Illumina Inc., San Diego, CA, USA) and 2 × 250 bp paired-end reads were generated.

The raw sequence data were processed using QIIME (www.qiime.org). Sequences with ≥97% similarity were assigned to the same operational taxonomic unit (OTU). All the reads were identified and classified using the UCHIME algorithm (42), compared against the reference database. To calculate the α-diversity of rumen microbiota, the following metrics were calculated: number of observed species, Chao1 index, and Shannon index (43). Principal coordinate analysis (PCoA) of weighted UniFrac distances, as described by Fomenky et al. (44), was used to visualize microbiota association patterns, i.e., β-diversity. Taxonomical annotation was performed using Mothur v 1.41.1 (43) and by referring to Silva.nr.132 (45). The number of shared genera was visualized in Venn diagrams. The linear discriminant analysis effect size (LEfSe) was used to analyze the bacterial differences at the genus level among the three treatment groups.

### RNA extraction and transcriptome, gene expression, and functional analyses

Total RNA was isolated from nine rumen tissue samples of lambs at 42 days of age, using TRIzol (TransGen Biotech, Beijing, China), according to the manufacturer's instructions. The concentration and quality of total RNA were examined in a NanoDrop spectrophotometer (NanoDrop Technologies, Wilmington, DE, USA) and Bioanalyzer 2100 system (Agilent Technologies Inc.), respectively. After screening for RNA quality, a total of 1.5 μg RNA per sample was used to construct a cDNA sequencing library using the TruSeq Stranded mRNA Sample Prep Kit (Illumina Inc.), following the manufacturer's instructions (46). After library construction, all libraries were sequenced using the Illumina HiSeq 4000 platform (Illumina Inc.) at Shanghai Personalbio Science and Technology Co., Ltd. (Shanghai, China).

Low-quality reads and adapter sequences were removed using Trimmomatic (47). Subsequently, the high-quality reads were aligned to the sheep reference genome (*Ovis aries* v3.1) using TopHat2 (v2.0.13) and Bowtie2 (v2.3.3.1), as described previously (48, 49). Gene transcripts were assembled using String-Tie (v1.3.1) based on the reference genome model (50). Gene expression values were normalized to fragments per kilobase of transcript per million mapped (FPKM).

Differentially expressed genes (DEGs) in pair-wise comparisons (ES vs. Ctrl, ES vs. ES + EW, and ES + EW vs. Ctrl group) were identified using the DESeq R package (1.10.1) in R software (v3.5.1) (51). Adjusted *P* < 0.05 and absolute value of [log_2_ (fold change)] > 1.5 were both used as filter factors for identifying DEGs. KOBAS (v3.0) was used to identify DEGs in the Kyoto Encyclopedia of Genes and Genomes (KEGG) pathways (52).

### Quantitative real-time polymerase chain reaction

Four genes (transforming growth factor, *TGF*; and insulin like growth factor binding proteins 3, 5, and 6, *IGBP3, IGBP5*, and *IGBP6*) associated with the phosphatidylinositol 3 kinase-protein kinase B (PI3K-Akt) and TGF-β signaling pathways were employed to assess the relationship between ruminal morphology and gene expression. Another five genes (sodium-hydrogen exchangers 2 and 3, *NHE2* and *NHE3*; DR alpha, *DRA*; 3-hydroxy-3-methylglutaryl-CoA lyase; *HMGCL*, and monocarboxylate transporter 1; *MCT1*) associated with mineral absorption and carbohydrate metabolism were selected for correlation analysis with ruminal pH and total VFAs (TVFA).

Total RNA was extracted from the rumen tissues of 18 lambs at 42 days of age using TRIzol (TransGen Biotech). Absorbance at 260 and 280 nm was measured using a NanoDrop^Ⓡ^ ND-1000 spectrophotometer (Thermo Fisher Scientific) to assess RNA purity and yield, and reverse-transcribed into cDNA, following the manufacturer's instructions. The primers used (Table 2) were designed in Primer Premier 5.0 software (PREMIER Biosoft International, Palo Alto, CA, USA). The quantitative real-time PCR (qRT-PCR) was performed on an ABI3700 Real-Time PCR system (Applied Biosystems Inc., Foster City, CA, USA) under the following conditions: denaturation at 94 °C for 3 min; then, 40 cycles at 94 °C for 30 s, annealing (different temperature for each gene, Table 2) for 30 s, extension at 72 °C for 30 s; final extension at 72 °C for 10 min. Each 20 μL qRT-PCR reaction contained 50 ng cDNA, 10 μL of SYBR Green (TransGen Biotech), 0.4 μL of each primer, and 8.2 μL ddH_2_O. Beta actin was used as the internal control gene. The 2^−Δ*ΔCT*^ method was used to analyze the data (52).

**Table 2:** QPCR Primers used in the validation of DEGs of rumen tissue between *Hu* lambs with different early feeding strategies.

**Gene**	**Gene Name**	**GenBank accession no**.	**Primer Sequences (5'**−**3')**	**Location on template**	**Amplicon Length (bp)**	**Temperature (**°**C)**
*TGFβ1*	Transforming Growth Factor Beta 1	NM_001009400.2	F: TGACCCACAGAGAGGAAATAGA	14:53732833-53732851	94	57
			R: AACCCGTTGATGTCCACTTGAA	14:53736067-53736089		
*IGFBP3*	Insulin Like Growth Factor Binding Protein 3	NM_001159276.1	F: TCAGCCTTGCGGCGTCTA	4:83695529-83695546	275	60
			R: TGTGGGCGAGGTGGGATT	4:83698731-83698748		
*IGFBP5*	Insulin Like Growth Factor Binding Protein 5	NM_001129733.1	F: GCTGAAGGCTGAGGCTGTGAA	2:233468360-233468380	308	57
			R: TCCCATACTTGTCCACGCACC	2:233466022-233466043		
*IGFBP6*	Insulin Like Growth Factor Binding Protein 6	NM_001134308.1	F: AGAGTAAGCCCCAAGCAG	3:142894866-142894883	159	59
			R: CACGGAGTCCAGATGTTT	3:142894568-142894589		
*NHE2*	Solute Carrier Family 9 Member A2	XM_604493.9	F: GACATCACTTTGCTCCAGAATC	3:105570477-105570498	153	57
			R: CACTGTCACGGCGTCATTCA	3:105570345-105570365		
*NHE3*	Solute Carrier Family 9 Member A3	XM_042233997.1	F: TGTTCGGCAGCCTGATTG	16:78114481-78114499	225	59
			R: CACCACGAAGAAGGACACTA	16:78116667-78116685		
*DRA*	Solute Carrier Family 26 Member 3	NM_001184899.1	F: TACAGGAATCGTGGGCTAT	20:27397471-27397490	280	57
			R: TCTGGAGGAACATTGGTG	20:27398452-27398470		
*HMGCL*	3-Hydroxy-3-Methylglutaryl-CoA Lyase	XM_004005125.4	F: GCTCCACGAGACGGACTACAA	2:258630811-258630831	282	60
			R: CTCAGAGGCGGCTCCAAAGAT	2:258635813-258635833		
*MCT1*	Solute Carrier Family 16 Member 1	NM_003051.2	F: CTTGCCTTCAACTTAAATCCG	1:95078951-95078971	206	58
			R: TGCTTACTCTTGCCATAA	1:95078466-95078483		
*β-Actin*	Beta-tubulin	AF035420.1	F: TCCGTGACATCAAGGAGAAGC	24:39622009-39622029	186	58
			R: CCGTGTTGGCGTAGAGGT	24:39622347-39622364		

### Statistical analysis

Statistical analyses of growth performance, ruminal anatomy, morphology, and fermentation were performed in R (v3.5.1; Free Software Foundation, Boston, MA, USA), using the linear model:


$${\text{Y}}_{\text{ijk}}=\mu +{\text{T}}_{\text{i}}+{\text{A}}_{\text{j}}+{\left(\text{T}:\text{ A}\right)}_{\text{ij}}+\mu_{\text{k}}+{\text{BW}}_{\text{k}}+{\text{DR}}_{\text{k}}+\varepsilon_{\text{ijk}},$$

where Y_ijk_ is the value measured in treatment i at age j of lamb k; μ is the overall mean; T_i_ is the fixed effect of the three treatments (Ctrl, ES, and ES + EW; i = 1, 2, and 3), A_j_ is the fixed effect of age over the seven periods (days 7, 14, 28, 42, 56, 70, and 84; j = 1, 2,…,7); (T:A)_ij_ is the fixed effect of the interaction between treatment and age; μ_k_ is the random effect of different early rearing in lambs (k = 1, 2, 3,…18); BW_k_ and DR_k_ are the covariates “birth weight” and “diarrhea rate,” respectively; ε_ijk_ is the random residual error. A significant effect of the treatment was established at *P* < 0.05.

Bacterial abundance and gene expression data were analyzed using one-way analysis of variance (ANOVA) in SPSS Statistics 23.0 (IBM Co. Ltd., Chicago, IL, USA). The model was the following:


$${\text{Y}}_{\text{ij}}=\mu +{\text{T}}_{\text{i}}+\varepsilon_{\text{ij}}$$

where Y_ij_ is the dependent variable (j = 1, 2…,6); μ is the overall mean; T_i_ is the fixed effect of the three treatments (Ctrl, ES, and ES + EW; i = 1, 2, and 3); ε_ij_ is the random effect. A significant effect of the treatment was established at *P* < 0.05.

Spearman's rank correlation was employed to assess the relationship between rumen fermentation parameters, ruminal morphology, gene expression, and relative bacterial abundance using R (v3.5.1; Free Software Foundation). A significant effect of the treatment was established at *P* < 0.05. All data were analyzed visually using GraphPad Prism 8.0.1 (GraphPad Software, Inc., San Diego, CA, USA) and Origin software (Origin Lab Corp., Northampton, MA, USA).

## Results

### Growth performance

**Figure 2:**
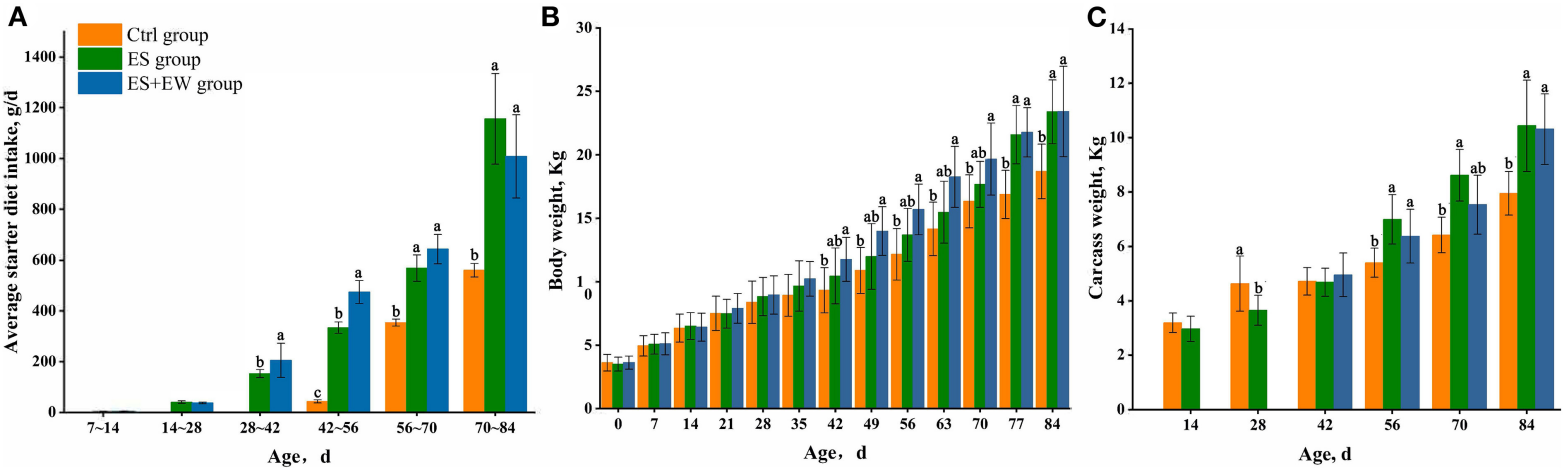
Average starter diet intake **(A)**, body weight **(B)**, and carcass weight **(C)** of lambs in different early feeding strategies. Columns with different letters at a single time point indicate means that differed based on the means separation (*P* < 0.05). Error bars indicate SEM for the treatment × age interaction.

The average starter diet intake of lambs was significantly affected by age, rearing, and their interaction (*P* = 0.022; Figure 2A). Lambs in the ES and ES + EW group were supplemented with starter diets until the end of the experiment (from 42 to 84 days of age), and their average starter diet intake was higher than that of lambs in the Ctrl group (*P* < 0.05). There was no significant interactive effect of rearing and age on the body and carcass weights of lambs (*P* > 0.05). After 42 days of age, the body weight of lambs in the ES + EW group was higher than that of lambs in the Ctrl group (*P* < 0.05; Figure 2B). The body weight of lambs in the ES group was higher than that of the lambs in the Ctrl group (*P* < 0.05) from 77 to 84 days of age (Figure 2B). The carcass weight of lambs in the ES and ES + EW groups was higher than that of the lambs in the Ctrl group after 56 days of age (*P* < 0.05, Figure 2C), except for lambs in the ES + EW group at 70 days of age (*P* > 0.05). Notably, the carcass weight of lambs in the Ctrl group was higher than that of lambs in the ES group (*P* < 0.05) at 28 days of age.

### Ruminal anatomy, morphology, and fermentation

**Figure 3:**
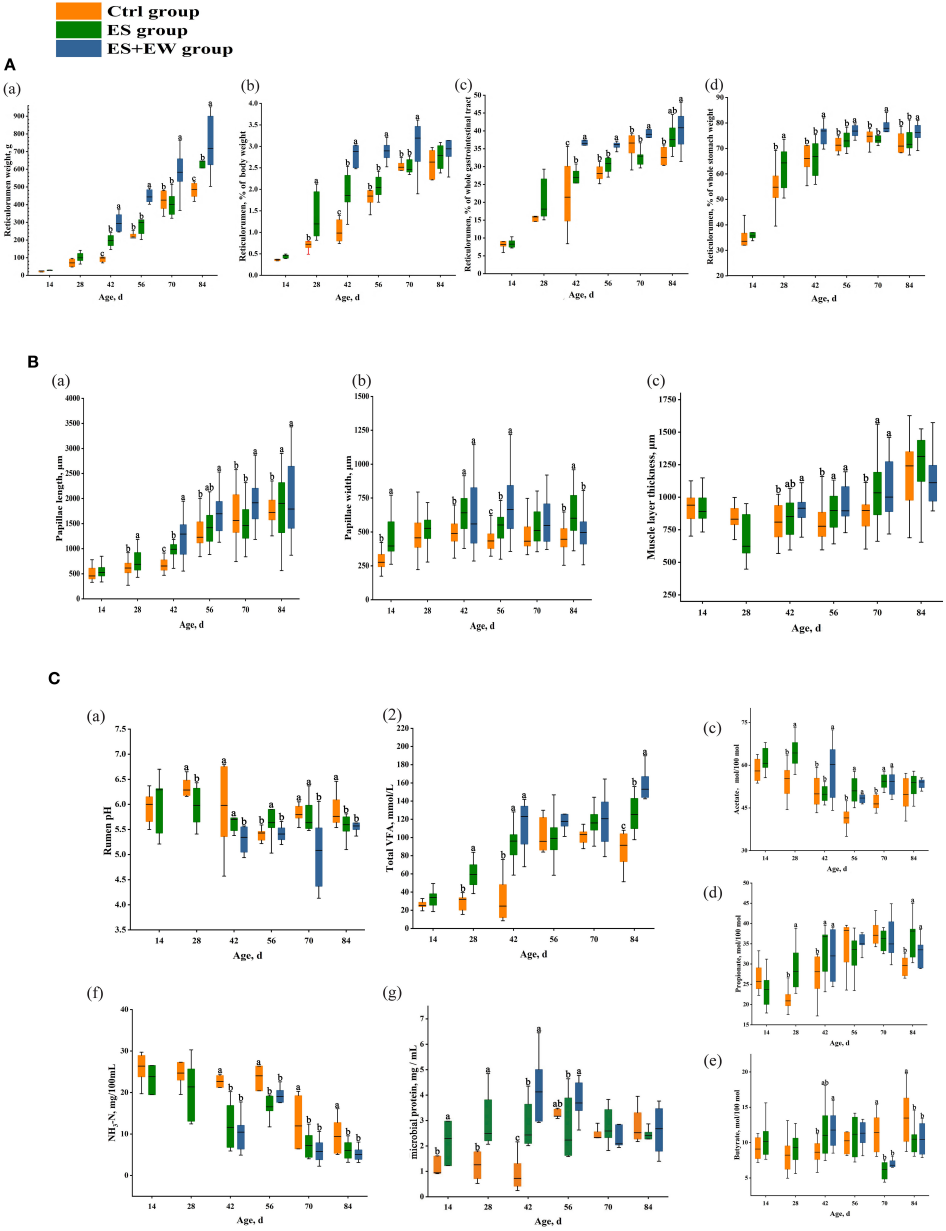
The development of lamb ruminal anatomy **(A)**, morphology **(B)**, and fermentation **(C)** in different early feeding strategies. Boxes with different letters at a single time point indicate means that differed based on the means separation (*P* < 0.05). Error bars indicate SEM for the treatment × age interaction.

The weights of the reticulorumen, which were expressed as a percentage of the body weight, whole gastrointestinal tract, and whole stomach were significantly affected by age, rearing, and their interaction (*P* < 0.001; Figure 3A). Moreover, the reticulorumen weight of lambs in the ES + EW group was higher (*P* < 0.05) than that of lambs in the other groups from 56 to 84 days of age. Lambs fed with starter diets had a higher reticulorumen weight, expressed as a percentage of body weight (*P* = 0.034) and whole stomach weight (*P* = 0.027) at 28 days of age. Additionally, early weaning increased the reticulorumen weight of lambs compared with lambs in the ES (*P* = 0.007) and Ctrl (*P* < 0.001) groups at 42 days of age.

Ruminal papillae length (*P* = 0.012; Figure 3Ba) and width (*P* = 0.030; Figure 3Bb), and ruminal muscle layer thickness (*P* < 0.001; Figure 3Bc) were all significantly affected by age, rearing, and their interaction. Lambs in the ES group had longer rumen papillae than lambs in the Ctrl group (*P* < 0.05) at 28, 42, and 84 days of age. The rumen papillae of lambs in the ES + EW group were longer than that of lambs in the other groups from 42 to 84 days of age (*P* < 0.05). Furthermore, the rumen papillae of lambs in the ES + EW group were longer than that of lambs in the Ctrl group at 28 and 42 days of age (*P* < 0.05). Additionally, the rumen papillae of lambs in the ES + EW group were wider than that of lambs in the Ctrl group at 42 and 56 days of age (*P* < 0.05). Similarly, lambs in the ES group had wider rumen papillae than lambs in the Ctrl group at 14, 42, and 56 days of age (*P* < 0.05). At 84 days of age, the rumen papillae of lambs in the ES group were wider than those of lambs in the other groups (*P* < 0.05). The rumen muscle layer of lambs in the ES and ES+EW groups was thicker than that of lambs in the Ctrl group at 56 and 70 days of age (*P* < 0.05). The lambs in the ES and ES + EW group had a thicker rumen muscle layer than that in the Ctrl group at 56 and 70 days of age (*P* < 0.05). Additionally, the rumen muscle layer of lambs in the ES + EW group was thicker than that of lambs in the Ctrl group at 42 days of age (*P* < 0.05).

Ruminal VFA concentrations (*P* < 0.001; Figure 3Ca), MCP (*P* < 0.001; Figure 3Cg), molar proportions of acetate (*P* < 0.001; Figure 3Cc), propionate (*P* < 0.001; Figure 3Cd), and butyrate (*P* < 0.001; Figure 3Ce) were all significantly affected by age, rearing, and their interaction. Conversely, neither ruminal pH (*P* = 0.282; Figure 3Ca) nor NH_3_-N (*P* = 0.464; Figure 3Cf) were significantly affected by age and rearing. However, the lambs in the ES and ES + EW groups had higher ruminal pH than the lambs in the Ctrl group at 28, 42, 70, and 84 days of age (*P* < 0.05) and the ruminal pH of lambs in the ES group was higher than that of lambs in the other groups at 56 days of age (*P* = 0.046). Conversely, the ruminal pH of lambs in the ES + EW group was higher than that in other groups at 84 days of age (*P* = 0.017). As for NH_3_-N concentration, lambs in the ES and ES + EW groups showed lower values than lambs in the Ctrl group from 42 to 84 days of age (*P* < 0.05).

Lambs in the ES group had higher ruminal VFA concentrations than lambs in the Ctrl group at 28 and 84 days of age (*P* < 0.05). At 42 days of age, lambs in the ES and ES + EW groups had higher ruminal VFA concentrations than that in the Ctrl group (*P* = 0.014). Lambs in the ES and ES + EW groups showed higher molar proportion of acetate than lambs in the Ctrl group at 28, 56, and 70 days of age (*P* < 0.05). At 42 days of age, the molar proportion of acetate in lambs in the ES + EW group was higher than that of lambs in the other groups (*P* = 0.027). The lambs in the ES and ES + EW groups showed higher molar proportions of propionate than the lambs in the Ctrl group at 28, 42, and 84 days of age (*P* < 0.05). At 70 and 84 days of age, lambs in the ES and ES + EW groups had lower molar proportions of butyrate than lambs in the Ctrl group (*P* < 0.05). The molar proportion of butyrate in lambs in the ES + EW group was higher than that of lambs in the Ctrl group (*P* = 0.043). Additionally, lambs in the ES + EW group had higher molar proportions of butyrate than lambs in the ES group at 56 days of age (*P* = 0.038). The lambs in the ES and ES + EW groups had higher MCP than the lambs in the Ctrl group from 14 to 42 days of age (*P* < 0.05).

### Ruminal microbiota diversity and community structure

**Figure 4:**
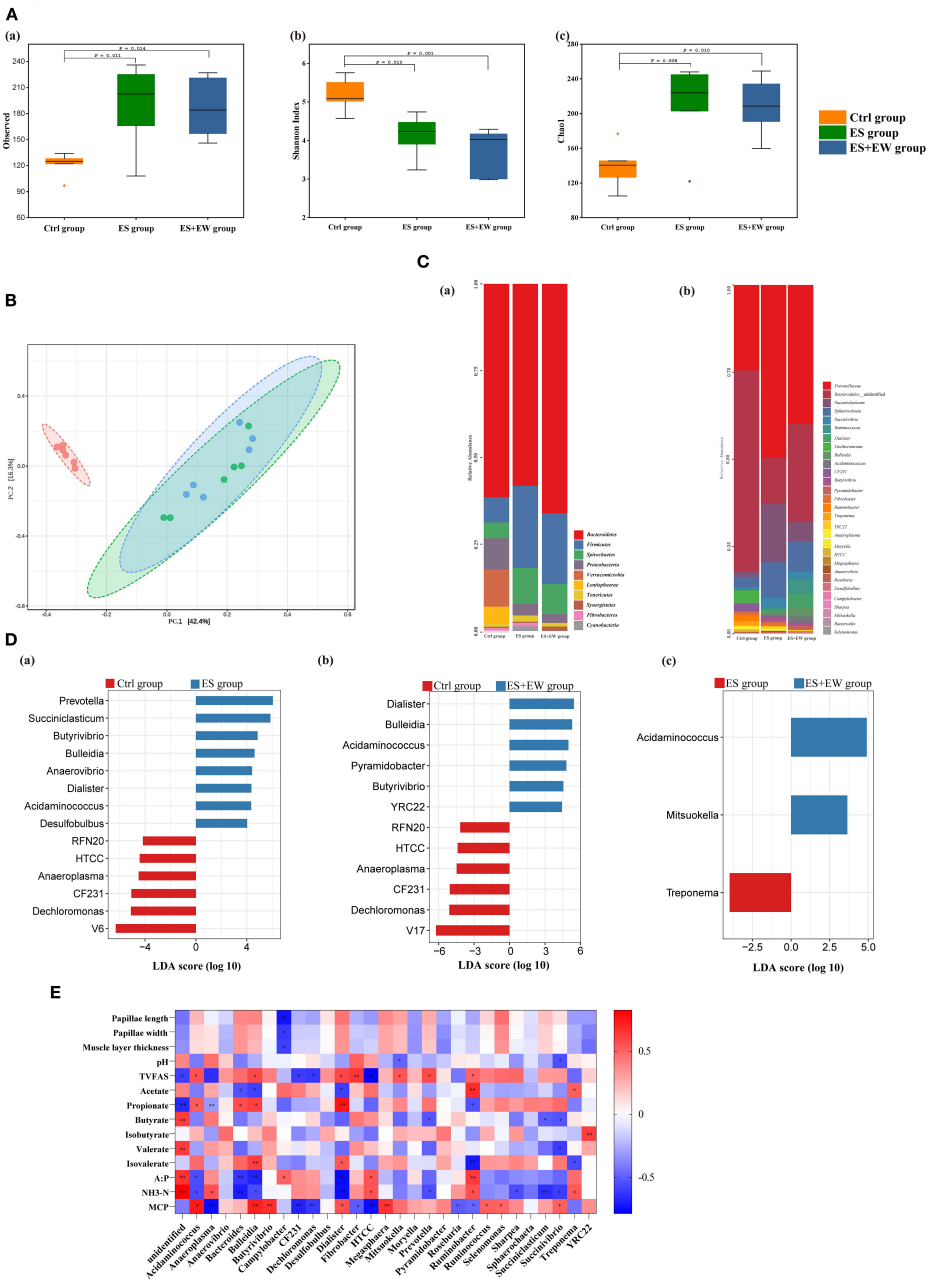
Ruminal microbiota diversity and community structure of lambs in different early feeding strategies at 42 days of age. **(A)** The alpha diversity in the rumen microbial community based on the observed species **(a)**, Shannon index **(b)** and Chao1 index **(c)**. **(B)** The principal coordinate analysis (PCoA) based on the unweighted Unifrac distances. **(C)** The composition of rumen microbiome at phylum and genus level: the composition of rumen microbiome at phylum level **(a)**, the composition of major rumen genera **(b)**. **(D)** LEfSe identified significantly different bacteria at the genus level as differentiating the two groups, including Ctrl vs. ES **(a)**, Ctrl vs. ES+EW **(b)**, and ES vs. ES+EW **(c)**. Genera in this graph were statistically significant (*P* < 0.05) and had an LDA Score > 2.5, which was considered a significant effect size; **(E)** Heat maps showing the correlations between animal phenotypical variables and relative abundance of bacterial genera. The depth of the color indicates the correlation between species and environmental factors. “*” and “**” indicate the different levels at 0.05 and 0.01, respectively.

A total of 810,363 high-quality sequences were obtained from the 18 samples of rumen contents, with an average of 45,020 sequences per sample, and 4,420 OTUs were defined based on 97% similarity. At 42 days of age, the number of observed species (*P* = 0.006; Figure 4Aa) and the value of Chao1 (*P* = 0.006; Figure 4Ac) in the Ctrl group were lower than that in the other groups. Conversely, the value of Shannon (*P* = 0.001; Figure 4Ab) was higher in the Ctrl group than in the other groups. However, no difference in α-diversity was observed between the ES and ES + EW groups (*P* > 0.05).

The results of the β-diversity analysis using weighted UniFrac distances revealed a clear diversification of the bacterial community between the Ctrl group and the other two groups (Figure 4B). The ANOSIM revealed differences in bacterial community composition between the Ctrl and ES groups (*R* = 0.572, *P* = 0.029) and the Ctrl and ES + EW groups (*R* = 0.681, *P* = 0.017). No significant differences were observed between the ES and ES + EW groups (*R* = 0.021, *P* = 0.641).

We identified the top ten most abundant phyla (Figure 4Ca) and the phyla with abundance > 1.00% of total sequences in at least one group. The main phylum, with the highest relative abundance across all samples, was Bacteroidetes, accounting for 58.19–66.06% of the total sequences. In the ES and ES + EW groups, it was followed by Firmicutes (23.65 and 20.45%, respectively), Spirochaetes (10.35 and 8.61%, respectively), and Proteobacteria (3.32 and 2.52%, respectively), while in the Ctrl group, it was followed by Verrucomicrobia (10.66%), Proteobacteria (8.84%), and Firmicutes (7.36%). The relative abundances of Proteobacteria (*P* = 0.007), Verrucomicrobia (*P* = 0.002), and Lentisphaerae (*P* = 0.014) were higher, whereas that of Firmicutes (*P* = 0.003) was lower in the Ctrl group than in the other groups. Bacterial genera with abundance > 0.10% are presented in Figure 4Cb. The lambs in the ES + EW group had a higher relative abundance of *Acidaminococcus* (*P* < 0.001), *Dechloromonas* (*P* = 0.021), high-throughput culture collection (HTCC) isolates (*P* = 0.002), *Mitsuokella* (*P* = 0.018), and *Roseburia* (*P* = 0.005) than in the other groups. *Bacteroidales*_unidentified (*P* = 0.002) and CF231 (*P* = 0.007) showed the highest relative abundance in the Ctrl group. The relative abundance of *Butyrivibrio* (*P* = 0.011) in the ES + EW group was higher than that in the Ctrl group. Lambs in the ES group had a higher relative abundance of *Succiniclasticum* (*P* = 0.020) than those in the Ctrl group.

To identify the differentiated bacterial taxa among the three rearing methods, LEfSe analysis was performed at the genus level. When the Ctrl and ES groups were compared (Figure 4Da), V6, *Dechloromonas*, CF231, *Anaeroplasma*, HTCC, and RFN20 were significantly enriched in the Ctrl group, whereas the biomarkers for the ES group were *Prevotella, Succiniclasticum, Butyrivibrio*, and *Bulleidia*. When the Ctrl and ES + EW groups were compared (Figure 4Db), V17, *Dechloromonas*, CF231, *Anaeroplasma*, HTCC, and RFN20 were enriched in the Ctrl group, whereas *Mitsuokella, Sharpea*, YRC22, *Butyrivibrio, Pyramidobacter, Acidaminococcus, Bulleidia*, and *Dialister* were biomarkers for the ES + EW group (Figure 4Db). Additionally, the rumen microbiota in lambs of the ES group had a higher relative abundance of *Treponema* than that of lambs in the other groups, whereas *Acidaminococcus* and *Mitsuokella* were over-represented in the EW + ES group (Figure 4Dc).

### Bacterial abundance and phenotypic variables relationship

Correlation analysis showed that ruminal muscle layer thickness (*R* = −0.596, *P* = 0.025) and papillae length (*R* = −0.726, *P* = 0.003), and width (*R* = −0.618, *P* = 0.018) were negatively correlated with the relative abundance of *Campylobacter* (Figure 4E; *n* = 6). Ruminal pH was negatively correlated with the relative abundances of *Mitsuokella* (*R* = −0.485, *P* = 0.041) and *Succinivibrio* (*R* = −0.546, *P* = 0.019). TVFA was positively correlated with the relative abundances of *Acidaminococcus* (*R* = 0.562, *P* = 0.024), *Bulleidia* (*R* = 0.593, *P* = 0.015), *Dialister* (*R* = 0.596, *P* = 0.015), *Mitsuokella* (*R* = 0.554, *P* = 0.026), and *Prevotella* (*R* = 0.585, *P* = 0.017) but negatively correlated with that of *Anaeroplasma* (*R* = −0.647, *P* = 0.007), CF231(*R* = −0.603, *P* = 0.013), *Dechloromonas* (*R* = −0.620, *P* = 0.010), *Fibrobacter* (*R* = −0.647, *P* = 0.007), HTCC (*R* = −0.786, *P* < 0.001), and *Ruminobacter* (*R* = −0.510, *P* = 0.043). The molar proportion of acetate was positively correlated with the relative abundances of *Ruminobacter* (*R* = 0.684, *P* = 0.003) and *Treponema* (*R* = 0.524, *P* = 0.037) but negatively correlated with that of *Bacteroides* (*R* = −0.539, *P* = 0.031), *Bulleidia* (*R* = −0.595, *P* = 0.015), and *Dialister* (*R* = −0.601, *P* = 0.014). The molar proportion of propionate was positively correlated with the relative abundances of *Acidaminococcus* (*R* = 0.527, *P* = 0.036), *Bacteroides* (*R* = 0.578, *P* = 0.019), *Bulleidia* (*R* = 0.616, *P* = 0.011), and *Desulfobulbus* (*R* = 0.735, *P* = 0.001) but negatively correlated with that of *Ruminobacter* (*R* = −0.536, *P* = 0.032). The molar proportion of butyrate was negatively correlated with the relative abundances of *Prevotella* (*R* = −0.526, *P* = 0.036), *Succiniclasticum* (*R* = −0.503, *P* = 0.047), and *Succinivibrio* (*R* = −0.531, *P* = 0.034). The molar proportion of isobutyrate was positively correlated with the relative abundance of YRC (*R* = 0.625, *P* = 0.010) while the molar proportion of valerate was negatively correlated with the relative abundance of *Succinivibrio* (*R* = −0.599, *P* = 0.014). The molar proportion of isovalerate was positively correlated with the relative abundances of *Bulleidia* (*R* = 0.624, *P* = 0.010) and *Dialister* (*R* = 0.542, *P* = 0.030), but negatively correlated with that of *Ruminobacter* (*R* = −0.716, *P* = 0.002) and *Treponema* (*R* = −0.565, *P* = 0.023). The ratio of acetate to propionate was positively correlated with *Campylobacter* (*R* = 0.505, *P* = 0.046), HTCC (*R* = 0.559, *P* = 0.024), and *Ruminobacter* (*R* = 0.635, *P* = 0.008) relative abundances but negatively correlated with *Acidaminococcus* (*R* = −0.550, *P* = 0.027), *Bacteroides* (*R* = −0.626, *P* = 0.009), *Bulleidia* (*R* = −0.684, *P* = 0.003), and *Dialister* (*R* = −0.760, *P* = 0.001) relative abundances. Ruminal NH_3_-N was positively correlated with the relative abundances of *Anaeroplasma* (*R* = 0.561, *P* = 0.019), HTCC (*R* = 0.559, *P* = 0.020), *Ruminobacter* (*R* = 0.526, *P* = 0.019), and *Treponema* (*R* = 0.545, *P* = 0.024) but negatively correlated with that of *Acidaminococcus* (*R* = −0.564, *P* = 0.018), *Bacteroides* (*R* = −0.704, *P* = 0.002), *Bulleidia* (*R* = −0.559, *P* = 0.020), *Dialister* (*R* = −0.744, *P* = 0.001), *Prevotella* (*R* = −0.549, *P* = 0.022), *Ruminobacter* (*R* = −0.562, *P* = 0.019), *Sharpea* (*R* = −0.555, *P* = 0.021), *Succiniclasticum* (*R* = −0.640, *P* = 0.006), and *Succinivibrio* (*R* = −0.600, *P* = 0.011). Ruminal MCP concentration was positively correlated with the relative abundances of *Acidaminococcus* (*R* = 0.745, *P* < 0.001), *Bulleidia* (*R* = 0.715, *P* = 0.003), *Butyrivibrio* (*R* = 0.666, *P* = 0.003), *Dialister* (*R* = 0.561, *P* = 0.015), *Megasphaera* (*R* = 0.660, *P* = 0.003), *Ruminococcus* (*R* = 0.516, *P* = 0.029), *Selenomonas* (*R* = 0.502, *P* = 0.034), and *Succinivibrio* (*R* = 0.514, *P* = 0.029) but negatively correlated with that of *Anaeroplasma* (*R* = −0.756, *P* < 0.001), CF231 (*R* = −0.715, *P* = 0.001), *Dechloromonas* (*R* = −0.686, *P* = 0.002), *Fibrobacter* (*R* = −0.619, *P* = 0.006), HTCC (*R* = −0.504, *P* = 0.033), *Roseburia* (*R* = −0.754, *P* < 0.001), and *Ruminobacter* (*R* = −0.507, *P* = 0.032).

### Characterization of the transcriptome in the rumen tissue

**Figure 5:**
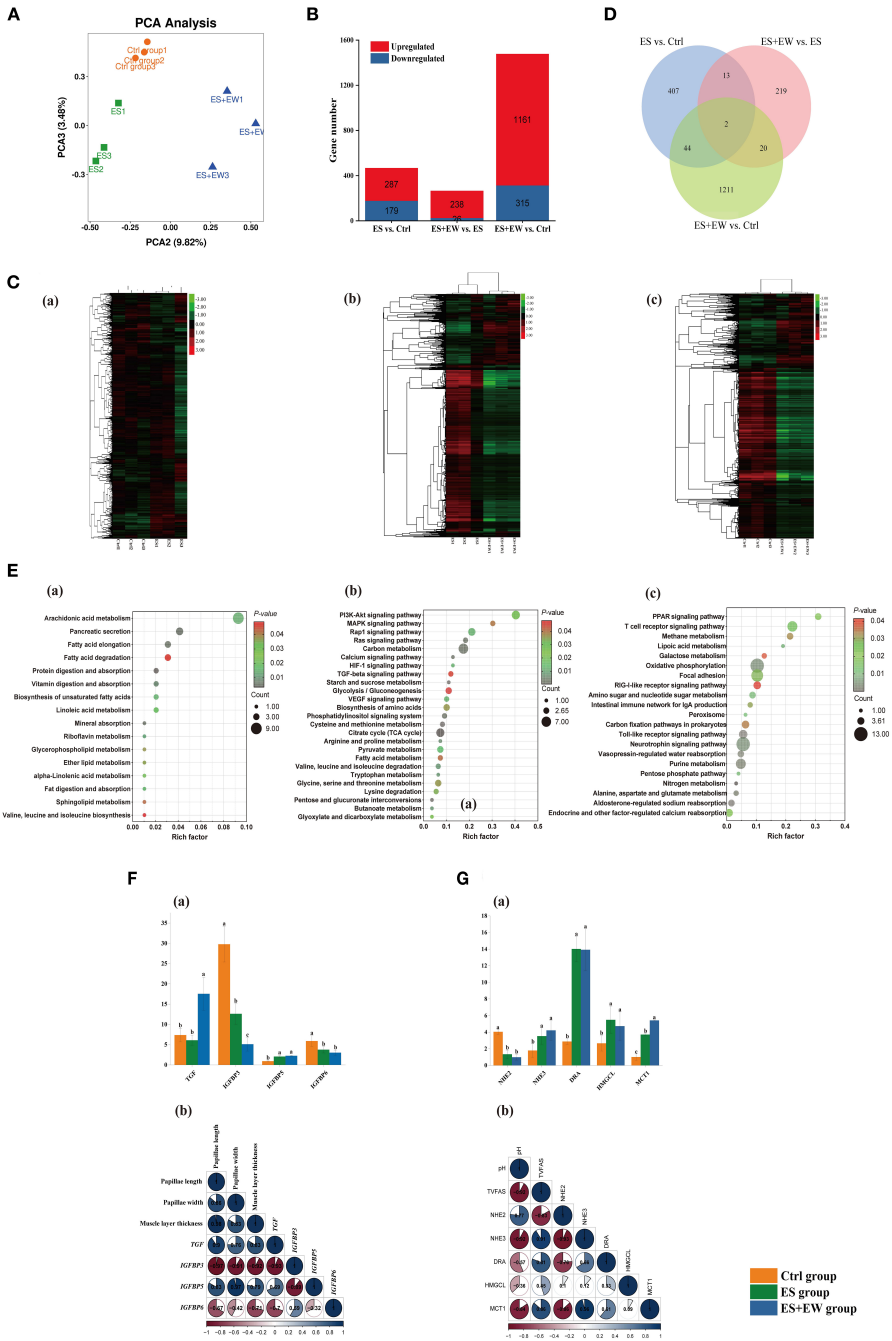
The PCA analysis of gene expressions, number of DEGs, KEGG pathways, and main DEGs in the rumen tissue of lambs in different early feeding strategies at 42 days of age. **(A)** The PCA analysis of gene expressions in the rumen tissue among three groups. **(B)** The number of DEGs at each comparison in the different early feeding strategies. **(C)** Clustering heat-map of the DEGs, including Ctrl vs. ES **(a)**, ES vs. ES+EW **(b)**, and Ctrl vs. ES+EW **(c)**; **(D)** The Venn diagram of all the comparisons and the numbers of DEGs at different early feeding strategies. **(E)** The KEGG pathways significantly enriched in the unique DEGs identified in the rumen tissue between Ctrl vs. ES **(a)**, ES vs. ES+EW **(b)**, and Ctrl vs. ES+EW **(c**), and DEGs in main KEGG pathways. The significance of identified KEGG pathways was determined by *P* < 0.05. **(F,G)** The relationship between DGEs expression and phenotypic variables, including gene expression related to the development of rumen epithelial morphology of lambs in three groups, *n* = 6 **(F**a**)**. Correlation heatmap showing the correlation of rumen epithelial morphology and DGEs, *n* = 6 **(F**b). Gene expression related to rumen fermentation of lambs in three groups, *n* = 6 **(G**a**)**; Correlation heatmap showing rumen fermentation correlated with DGEs, *n* = 6 **(G**b**)**. The qRT-PCR measurements of the expression of DGEs were analyzed using 2^−Δ*ΔCT*^ method.

A total of 467.71 million (51.97 ± 1.77 million reads per sample) high-quality, paired reads were obtained from nine rumen tissue samples using RNA sequencing, and the alignment rate to the *Ovis aries* reference genome was 89.01% (from 92.19% to 86.08%). Principal component analysis (PCA) of total gene expression demonstrated marked clustering among the three groups (Figures 5A,C). When compared with the Ctrl group, there were 466 genes that were differentially expressed in the ES group, including 287 upregulated and 179 downregulated genes (Figure 5B). Compared with the ES group, there were 264 DEGs in the ES + EW group, including 238 upregulated and 26 downregulated genes (Figure 5B). Additionally, there were 1,476 DEGs in the ES + EW group compared with the ES group, including 1,161 upregulated and 315 downregulated genes (Figure 5B).

### Functional analysis of DEGs

Compared with the Ctrl group, 407 unique DEGs were observed in the rumen tissue of the ES group (Figure 5D). The results of the KEGG pathway analysis showed that 16 pathways were significantly enriched (Figure 5Ea, *P* < 0.05). Most of these pathways were associated with nutrient transport and lipid metabolism, including arachidonic acid metabolism (including genes *PTGES, CBR1, ALOX12B, PLA2G, SPLA2*, and *AKR1C3*), fatty acid elongation (including genes *HADH, ELOVL7, HSD17B12, KAR*, and *IFA38*), fatty acid degradation (including genes *ACADS, ACSBG, HADH, ACSL, fadD*, and *ADH1_7*), and mineral absorption (including genes *SLC26A3, DRA*, and *CLCN2*).

Compared with the ES + EW group, 219 unique DEGs were observed in the rumen tissue of the ES group (Figure 5D). The KEGG pathway analysis revealed 25 significantly enriched pathways (Figure 5Eb, *P* < 0.05), most of which associated with cell proliferation, apoptosis, and differentiation, such as the PI3K-Akt (including genes *PTK2, FAK, PPP2R5, ITGB1, PPP2R1*, and *ITGB7*), MAPK signaling (including genes *RPS6KA, RSK2, PPM1B, PP2CB*, and *MAPK1_3*), and TGF-β (including genes *TFDP1* and *MAPK1_3*) pathways.

Additionally, compared with the Ctrl group, 1211 unique DEGs were observed in the rumen tissue of the ES + EW group (Figure 5D). The KEGG pathway analysis showed that 21 pathways were significantly enriched (Figure 5Ec, *P* < 0.05), including the T-cell receptor signaling pathway (including genes *PPP3R, CNB, NFKB1, FOS, JUN, MAPK1_3, DLG1*, and *RHOA*), the intestinal immune network for immunoglobulin A (IgA) production (including genes *CXCR4, TNFRSF3*, and *LTBR*), toll-like receptor signaling pathway (including genes *MAPK1_3, JUN, FOS, MAP2K3, MKK3*), methane metabolism (including genes *ACSS, PGAM*, and *gpmA*), and galactose metabolism (including genes *UGP2, galU, galF*, and *GAA*). These pathways are also associated with immune functions and carbohydrate metabolism.

### Relationship between DEGs and phenotypic variables

Figure 5F shows the relationship between rumen morphology and DEGs (n = 6). Rumen muscle layer thickness (*R* = 0.831, *P* = 0.002; *R* = 0.694, *P* = 0.035; *R* = −0.920, *P* = 0.001; *R* = −0.705, *P* = 0.021), and papillae length (*R* = 0.895, *P* < 0.001; *R* = 0.826, *P* = 0.002*; R* = −0.966, *P* < 0.001; *R* = −0.673, *P* = 0.032) and width (*R* = 0.757, *P* = 0.001; *R* = 0.967, *P* < 0.001; *R* = −0.907, *P* = 0.001) were positively correlated with *TGF* and *IGFBP5* expression, but negatively correlated with *IGFBP3* and *IGFBP6* expression. Spearman's rank correlation analysis was used to explore the relationship between rumen fermentation and DEGs (Figure 5G, n = 6). The expression of *NHE3* (*R* = −0.920, *P* < 0.001; *R* = 0.905, *P* = 0.001), *DRA (R* = −0.573, *P* = 0.046; *R* = 0.812, *P* = 0.017) and *MCT1* (*R* = −0.844, *P* = 0.004; *R* = 0.862, *P* = 0.003) was negatively correlated with ruminal pH but positively correlated with rumen TVFA concentration. Conversely, *NHE2* (*R* = 0.767, *P* = 0.009; *R* = −0.826, *P* = 0.002) expression was positively correlated with ruminal pH but negatively correlated with rumen TVFA concentration.

## Discussion

### Growth performance

Rumen development in pre-weaning young ruminants influences their adaptation from liquid to solid diets and post-weaning growth performance (53). The present study showed that the average starter diet intake of lambs in the ES + EW group (weaned at 28 days of age) was higher than that in other groups from 28 to 56 days of age. Furthermore, as the lambs were provided with a starter diet at 7 days of age, there was no rapid reduction in the average starter diet intake and body weight (from 28 to 35 days of age) of lambs weaned at 28 days of age. The separation of lambs from their dams while the latter were fed (and the lambs were supplemented) might thus have helped the lambs to successfully adapt to the abrupt separation from their dams at 28 days of age. Such conditions might have favored a smooth transition from milk to solid diets and enabled the lambs to be completely dependent on a solid diet as their source of protein and carbohydrates. However, these results contradict those reported by Carballo et al. (54) and Wang et al. (10). This discrepancy might be explained by the difference in the time at which starter diets were provided to lambs, weaning age, and the composition and nutritional levels of starter diets. A sharp increase in the average starter diet intake was observed from 56 to 70 days of age in lambs in the ES group (weaned at 56 days of age). After changing the diet, lambs in the ES group had a short-term (from 70 to 84 days of age) higher average starter diet intake, body weight, and carcass weight than lambs in the other groups. These results suggest that early life stress may have a long-lasting effect on the performance of lambs (55). It is worth noting that the carcass weight of lambs in the Ctrl group was higher than that of lambs in the other groups at 28 days of age. This might be explained by young ruminants relying solely on the nutrients obtained from milk during the first four weeks of life (53).

### Ruminal anatomy, morphology, and fermentation

The anatomical and physiological development of the rumen is one of the most important events during the weaning transition of a young ruminant (56). Previous studies by Baldwin et al. (29) and Diao et al. (9) on rumen development in the pre- and post-weaning stages have shown that at birth, 8 weeks, and 12–16 weeks of age the reticulorumen accounts for 38%, 61.23%, and 67% of the entire stomach weight, respectively. These observations are consistent with those of the present study. Furthermore, early weaning and provision of starter diets to lambs promoted rumen development, which is in accordance with the results of previous studies showing that providing solid feed as early as possible is beneficial to the rapid development of the rumen (20). The physical development of the rumen can be divided into two aspects: an increase in muscle mass and the growth of the papillae. Steele et al. (57) and Aschenbach et al. (58) suggested that the ruminal epithelium plays an important role in ruminal development, including absorption, transport, short-chain fatty acid metabolism, and protection. Similarly, Lesmeister et al. (40) found that ruminal epithelium length was the most important factor for evaluating rumen development, followed by ruminal epithelium width and muscle layer thickness. In the present study, feeding lambs with starter diets stimulated ruminal morphological development, and this effect was more effectively promoted by early weaning. Stimulation of ruminal physical development is determined by the nutritional composition and physical structure of the starter diets fed to lambs (17). In particular, the ruminal epithelium of lambs in the Ctrl group was shorter than that of lambs in the other groups after diet transition (from 70 to 84 days of age), further supporting that early nutritional regulation may have long-term effects on the performance of lambs.

Rumen pH is essential for rumen development, rumen environment, and even lamb health. The ruminal pH of lambs has been reported to be lower than that of adult sheep (59), which is similar to our present results. The lambs in the present study showed no signs of metabolic disorders, suggesting that lambs at this age tolerate pH levels that would be detrimental to the health of adult sheep. In addition, as lambs consumed more of the starter diet, rumen digesta pH decreased, whereas VFA concentrations gradually increased. Liquid diets limit metabolic activity and VFA absorption in the rumen epithelium (60), retarding development. It is noteworthy that early weaning contributes to rapid rumen development, the positive effect of which was maintained for up to 2 weeks after diet transition. Our study found higher molar proportions of acetate in the rumen of lambs fed with starter diets, especially in the EW + ES group. This result was similar to that reported by Terré et al. (61), and might be due to the higher starter diet intake in the EW + ES group, which promoted rumen development. The present study also showed that NH_3_ concentrations decreased with the increasing age of lambs, which concurs with the results of previous studies (62, 63). The lambs in the ES and EW + ES groups had higher ruminal NH_3_-N concentrations than lambs in the Ctrl group from 42 to 84 days of age. The subsequent decrease in rumen NH_3_-N concentration is possibly due to better utilization of NH_3_ by rumen microorganisms and to the dilution effect arising from a greater rumen volume (64). Microbial proteins play an important role in supplying protein to ruminants and provide most of the amino acids needed for the growth, maintenance, and production of the host animal (65). In the present study, lambs in the Ctrl group had lower MCP levels than lambs in the other groups, confirming that feed intake affects the rate of microbial protein synthesis (66). In summary, starter fermentability and intake play an important role in rumen development.

### Rumen bacterial diversity and community structure

The α- and β-diversity of the bacterial community structure of lambs in the Ctrl group were distinct from those of lambs in the other groups. In the present study, early supplementation and weaning caused a decrease in microbial diversity, which is consistent with the results of a previous study (18). Solid diets are the main factor influencing rumen bacterial community structure. The important turning point in microbial colonization is the introduction of a solid diet to young ruminants (67). Kim et al. (22) reported that feeding Holstein calves starter diets with forage was conducive to an increase in microbial richness. As so, the results of the present study are mostly consistent with those of previous studies (18, 68, 69), with slight differences. These differences may be related to the different feeding strategies. In the present study, the rumen microbiota was dominated by Bacteroidetes, Firmicutes, and Proteobacteria, similar to the observations of previous studies (18, 70). The relative abundance of Proteobacteria in the Ctrl group was higher than that in the other groups. Yáñez-Ruiz et al. (71) concluded that the establishment of ruminal bacterial communities in lambs from birth to weaning is rapid, with Proteobacteria being gradually replaced by Bacteroidetes as the main phylum. In ruminants, Firmicutes play an important role in degrading fiber (22), while the main function of Bacteroidetes is to degrade carbohydrates and proteins, which can encourage the functional development of gastrointestinal immunity (72). In the present study, lambs in the Ctrl group had a lower abundance of Bacteroidetes than lambs in the other groups. Malmuthuge et al. (73) reported that providing a starter diet could propel rumen microbiota development to a more mature status. The abundance of Verrucomicrobia was second only to Bacteroidetes in the Ctrl group and was substantially higher than that in the other groups. The present results are also consistent with those of a previous study (74). Verrucomicrobia have been suggested to coevolve with the mammalian gut, as this phylum plays an important role in maintaining gut homeostasis. Shen et al. (75) reported that Verrucomicrobia modulate the expression of pathways related to the immune tolerance of the rumen epithelium. At the genus level, the abundance of *Acidaminococcus* was higher in the ES + EW group than in the other groups. *Acidaminococcus* is a genus in the phylum Firmicutes, and its members are anaerobic diplococci that can use amino acids as their sole energy source for growth. *Acidaminococcus* may be responsible for the increased metabolism of amino acids, carbohydrates, and lipids (76). According to the dietary intake analysis of the three groups, the higher intake of starter diets containing high levels of carbohydrates and amino acids after early weaning probably provided more nutrients for metabolism and growth of *Acidaminococcus*. Additionally, lambs in the ES + EW group had higher *Mitsuokella* abundance than lambs in the other groups. Zhang et al. (77) found that *Mitsuokella* abundance was positively correlated with dry matter intake in calves, similar to the present results. Lambs fed with solid diets before weaning had a greater abundance of *Prevotella*. McLoughlin et al. (78) indicate that *Prevotella* is driven by dietary composition and independent of weaning. Our results support previous observations showing that the inclusion of starter concentrate in the diet of pre-weaning lambs promotes rumen colonization by *Succiniclasticum* (79). In short, feeding pre-weaning lambs with starter diets can change the structure of the rumen bacterial community and contribute to convergence toward the microbiota profile of adult animals. Early weaning increases the starter intake of lambs, thereby stimulating rumen microbial colonization.

### Molecular mechanism of early weaning and effect of early supplementation on ruminal morphology and functional development based on transcriptome analysis

Compared with the Ctrl group, 407 unique genes were differentially expressed in the ES group. The enriched KEGG pathways were mainly related to nutrient transport and metabolism in rumen tissue. Fatty acids are essential for cell proliferation in the cellular membranes (80). According to our findings, the upregulated genes *HADH, HSD17B12, KAR, IFA38, ACADS, ACSL, fadD*, and *ADH1_7* were enriched in the fatty acid elongation and degradation pathway, which is beneficial to the proliferation and turnover of rumen epithelial cells. Previous studies have identified that these genes are associated with fatty acid synthesis and deposition (81–83). This may have contributed to the higher carcass weight of lambs in the ES group than in the Ctrl group from 56 to 84 days of age. Moreover, arachidonic acid metabolism is a significantly enriched signaling pathway in ruminal tissue of lambs fed with solid diets (84), which is consistent with our results. It is well established that arachidonic acid regulates cellular inflammation, oxidative stress, proliferation, and membrane permeability (85), which may affect rumen immune function. In the present study, the expression of *SLC26A3* and *DRA* was increased in the ES group, and enriched in ion and mineral absorption pathways, which has been shown to be conducive to ruminal absorption of VFA (27). Taken together, these results suggest that early supplementation may enhance nutrient metabolism and transport in pre-weaned lambs.

Functional analysis of the 219 unique DEGs in the ES + EW vs. ES groups showed that early weaning affected cell proliferation, apoptosis, and differentiation. Compared with lambs in the ES group, 13 upregulated genes (*PTK2, FAK, PPP2R5, ITGB1, PPP2R1, RPS6KA, RSK2, PPM1B, PP2CB, CALM, CTNNB1, TFDP1* and *MAPK1_3*) and one downregulated gene (*ITGB7*) were enriched in the PI3K-Akt, MAPK, Rap1, Ras, and TGF-β signaling pathways of ES + EW lambs. All of these genes contribute to the establishment of the rumen epithelial function and barrier (86, 87). Notably, we also identified many signaling pathways involved in amino acid metabolism. Previous studies have suggested that a comparatively low number of amino acids and peptides may be absorbed and metabolized by the rumen tissue (88). However, the development of the rumen epithelium requires increased cell and protein turnover (25, 89). In the present study, many upregulated DEGs in the ES + EW group were annotated in pathways related to amino acid metabolism, including biosynthesis of amino acids, cysteine and methionine metabolism, arginine and proline metabolism, valine, leucine, and isoleucine degradation; glycine, serine, and threonine metabolism; and lysine degradation. In summary, we hypothesize that MCP synthesis might promote protein turnover and oxidation in the rumen. These metabolites are substrates for the citric acid cycle, generate energy-containing compounds, are responsible for the activation of some biological processes, and might further promote rumen epithelium development (84). Therefore, early weaning increases the intake of solids in lambs, promoting nutrient transport, degradation (especially of nitrogenous substances), and absorption in the rumen epithelium.

Compared to the Ctrl group, 1,121 unique genes were differentially expressed in the ES + EW group. These genes were mainly enriched in the biological processes of immune function and nutrient metabolism, which play important roles in rumen development. Naeem et al. (80) suggested that the peroxisome proliferator-activated receptor (PPAR) signaling pathway could be important in promoting rumen metabolism and development. The PPAR signaling pathway was the most significantly altered pathway induced by the interaction between early supplementation and early weaning. Toll-like receptors (TLRs) play a critical role in suppressing inflammation in the gastrointestinal epithelium by reducing the production of inflammatory cytokines. Shen et al. (75) found that the carbohydrates in the starter diet induced the expansion of the rumen microbiota and promoted epithelium tolerance by enhancing the intensity of toll-like signaling, and the newly established equilibrium benefited the transport of ruminal energy substances into the blood. Similarly, during the analysis of shared DEGs in the toll-like signaling pathway, the expression of toll-like signaling pathway genes in the ES + EW group was lower than that in the Ctrl group. Taking these results together, we hypothesize that there is better resistance to inflammation and a greater ability to transport energy substances within the rumen tissue of ES + EW lambs.

Hayashi et al. (90) indicated that insulin-like growth factor 1 (IGF1) can stimulate epithelial cell proliferation and differentiation to enhance ruminal papillae development. IGF1 induces a cellular response by regulating IGFBPs. When *IGFBP5* is upregulated, it potentiates IGF1 effects, stimulating the proliferation of rumen epithelium, whereas *IGFBP3* regulates this process in the opposite direction (91). In the present study, early supplementation increased *IGFBP5* expression and decreased *IGFBP3* expression, which should promote rumen epithelial cell proliferation to facilitate the development of ruminal morphology. NHEs are apical membrane sodium ion (Na^+^)-hydrogen ion (H^+^) anti-porters involved in the regulation of intracellular pH. The expression of *NHEs* is upregulated by butyrate; however, the long-term regulation of *NHEs* is primarily accomplished by changes in transcription (92). In the present study, the mRNA expression of *NHE2* was higher in the Ctrl group than in the other groups, while *NHE3* expression was lower in the Ctrl group than in the other groups. *NHE3* imports Na^+^ to the cell and exports H^+^ to the rumen, while *NHE2* imports H^+^ to the cell and exports Na^+^ to the extracellular space (27). Therefore, the lower *NHE2* expression and higher *NHE3* expression may demonstrate decreased H^+^ recycling into the lumen and a greater net H^+^ uptake by the rumen epithelium to maintain a stable rumen environment. Schurmann et al. (93) proposed that DRA is responsible for neutralizing acid in the rumen by exporting bicarbonate ions from epithelial cells and importing dissociated VFAs. MCT1, which is localized in the basolateral membrane, is involved in the basolateral export of ketone bodies arising from butyrate metabolism and lactate arising from propionate metabolism (94). The expressions of *DRA* and *MCT1* in the Ctrl group were lower than that in the other groups, which concurs with the results of Laarman et al. (95). These results may explain why early supplementation leads to an increase in VFA concentration within the rumen.

## Conclusion

In summary, feeding starter diets to lambs is a turning point in rumen development, and it accelerates rumen development with increased feed intake after weaning. The present study indicated that feeding lambs with starter diets at 7 days of age and weaning them at 28 days of age can stimulate rumen morphological development, promote rumen microbial colonization, and improve rumen epithelial anti-inflammatory and nutrient transport capacities. The positive effects of this early feeding strategy on lamb development persisted for up to 2 weeks after the lambs changed to fattening diets. However, our study has only examined the short-term effects of early rearing practices after changing to fattening diets, and more work is necessary for understanding the long-term influence of early rearing on lambs.

## Data availability statement

The raw sequence data obtained in this study can be found on the NCBI Sequence Read Archive under accession numbers PRJNA594822 and PRJNA317746.

## Ethics statement

All experimental protocols and sample collection methods were approved by the Ethics Committee of Gansu Agriculture University under Permit No. 2012-2-159.

## Author contributions

FL and WW designed the study. TL, XWa, ZM, and CL conducted the experiment and sample collection. TL, WW, XWa, and ZM performed data analysis. TL and CZ produced the initial draft of the manuscript and contributed to the revision of the manuscript. XWe revised the manuscript and provided the experimental resources. All authors reviewed and approved the final manuscript.

## Funding

This study was supported by grants from the National Natural Science Foundation of China (31860656), the Youth Science and Technology Fund Program of Gansu Province (20JR10RA553), the Provincial Natural Fund of Gansu (20IR1ORA538), and the Special Talent Introduction Program of Gansu Agricultural University (GSAU-RCZX201710).

## Conflict of interest

The authors declare that the research was conducted in the absence of any commercial or financial relationships that could be construed as a potential conflict of interest.

## Publisher's note

All claims expressed in this article are solely those of the authors and do not necessarily represent those of their affiliated organizations, or those of the publisher, the editors and the reviewers. Any product that may be evaluated in this article, or claim that may be made by its manufacturer, is not guaranteed or endorsed by the publisher.
