# Supplementary material for: Early feeding strategies in lambs affect rumen development and growth performance, with advantages persisting for two weeks after the transition to fattening diets
Source: Front Vet Sci. 2022 Jul 28;9:925649. doi: 10.3389/fvets.2022.925649 (PMC9366302; doi:10.3389/fvets.2022.925649)
Supplement: Supplementary file 1 [file Table_1.DOCX]

Table S1 Ingredients and chemical composition of ewes’ diet (air-dry basis, %)

| Ingredients | Composition |
| --- | --- |
| Sunflower skin | 18.0 |
| Corn straw | 26.0 |
| Corn germ cake | 11.0 |
| Corn | 30.0 |
| Soybean meal | 8.0 |
| Corn gluten meal | 3.5 |
| Limestone | 0.2 |
| Calcium bicarbonate | 0.2 |
| Salt | 0.6 |
| Premix^1^ | 2.5 |
| Total | 100.0 |
| Chemical composition |  |
| Dry matter | 89.07 |
| Digestive energy MJ/kg | 10.98 |
| Crude protein | 12.65 |
| Ether extract | 2.14 |
| Neutral detergent fiber | 39.17 |
| Acid detergent fiber | 25.56 |
| Calcium | 0.94 |
| Total phosphorus | 0.31 |

^1^ The premix provides minerals and vitamins per kg: Fe 69.63 mg, Cu 69.63 mg, Mn 23.70 mg, Zn 55 mg, I 0.67 mg, Se 0.3 mg, Co 0.3 mg, Vitamin A 2500 IU.
